# Increased platinum accumulation in SA-1 tumour cells after in vivo electrochemotherapy with cisplatin

**DOI:** 10.1038/sj.bjc.6690222

**Published:** 1999-03

**Authors:** M C̆emaz̆ar, D Miklavc̆ic̆, J S̆c̆anc̆ar, V Dolz̆an, R Golouh, G Sers̆a

**Affiliations:** Institute of Oncology, University of Ljubljana, Zalos̆ka 2, Ljubljana, SI-1000 Ljubljana, Slovenia; Faculty of Electrical Engineering, University of Ljubljana, Trz̆as̆ka 25, Ljubljana, SI-1000 Ljubljana, Slovenia; Joz̆ef Stefan Institute, University of Ljubljana, Jamova 39, Ljubljana, SI-1000 Ljubljana, Slovenia; Medical Faculty, University of Ljubljana, Vrazov trg 2, Ljubljana, SI-1000 Ljubljana, Slovenia

**Keywords:** cisplatin, platinum, electroporation, electrochemotherapy, electrothermal atomic absorption spectrometry

## Abstract

Electrochemotherapy is an anti-tumour treatment that utilizes locally delivered electric pulses to increase cytotoxicity of chemotherapeutic drugs. The aim of our study was to determine whether anti-tumour effectiveness of electrochemotherapy with cisplatin is a consequence of increased plasma membrane permeability caused by electroporation that enables cisplatin binding to DNA. For this purpose, anti-tumour effectiveness of electrochemotherapy was evaluated on SA-1 tumours treated with electric pulses 3 min after intravenous injection of cisplatin (4 mg kg^−1^). Anti-tumour effectiveness was correlated with platinum accumulation in tumours and the amount of platinum bound to DNA, as determined by atomic absorption spectrometry. In tumours treated with electrochemotherapy, cell kill was increased by a factor of 20 compared with treatment with cisplatin only, as determined from tumour growth curves. The amount of platinum bound to DNA and platinum content in the tumours treated by electrochemotherapy was approximately two times higher than in cisplatin-treated tumours. Based on our results, we conclude that in vivo application of electric pulses potentiates anti-tumour effectiveness of cisplatin by electroporation that consequently results in cisplatin increased delivery into the cells. In addition, besides electroporation, immune system and tumour blood flow changes could be involved in the observed anti-tumour effectiveness of electrochemotherapy. © 1999 Cancer Research Campaign


*British Journal of Cancer* (1999) **79**(9/10), 1386–1391
© 1999 Cancer Research Campaign
Article no. bjoc.1998.0222

### Increased platinum accumulation in SA-1 tumour cells after in vivo electrochemotherapy with cisplatin

**M Čemažar[1], D Miklavčič[2], J Ščančar[3], V Dolžan[4], R Golouh[1] and G Serša[1]**

[1]Institute of Oncology, Zaloška 2, SI-1000 Ljubljana; [2]University of Ljubljana, Faculty of Electrical Engineering, Tržaška 25, SI-1000 Ljubljana; [3]Jožef Stefan Institute, Jamova 39, SI-1000 Ljubljana; [4]University of Ljubljana, Medical Faculty, Institute of Biochemistry, Vrazov trg 2, SI-1000 Ljubljana, Slovenia

**Summary** Electrochemotherapy is an anti-tumour treatment that utilizes locally delivered electric pulses to increase cytotoxicity of chemotherapeutic drugs. The aim of our study was to determine whether anti-tumour effectiveness of electrochemotherapy with cisplatin is a consequence of increased plasma membrane permeability caused by electroporation that enables cisplatin binding to DNA. For this purpose, anti-tumour effectiveness of electrochemotherapy was evaluated on SA-1 tumours treated with electric pulses 3 min after intravenous injection of cisplatin (4 mg $kg^{-1}$). Anti-tumour effectiveness was correlated with platinum accumulation in tumours and the amount of platinum bound to DNA, as determined by atomic absorption spectrometry. In tumours treated with electrochemotherapy, cell kill was increased by a factor of 20 compared with treatment with cisplatin only, as determined from tumour growth curves. The amount of platinum bound to DNA and platinum content in the tumours treated by electrochemotherapy was approximately two times higher than in cisplatin-treated tumours. Based on our results, we conclude that in vivo application of electric pulses potentiates anti-tumour effectiveness of cisplatin by electroporation that consequently results in cisplatin increased delivery into the cells. In addition, besides electroporation, immune system and tumour blood flow changes could be involved in the observed anti-tumour effectiveness of electrochemotherapy.

**Keywords:** cisplatin; platinum; electroporation; electrochemotherapy; electrothermal atomic absorption spectrometry

Electroporation is a physical method in which high voltage direct current electric pulses cause non-selective plasma membrane permeabilization. This method is widely used for introduction of molecules such as DNA, antibodies, enzymes, dyes and drugs into cells (Orlowski and Mir, 1993). The use of electric pulses to facilitate delivery of chemotherapeutic drugs into tumour cells was termed electrochemotherapy and was introduced by Okino (Okino and Mohri, 1987) and Mir (Mir et al, 1991). Anti-tumour effectiveness of electrochemotherapy with the chemotherapeutic drugs bleomycin and cisplatin has been extensively elaborated on various tumour models in vivo (Serša et al, 1994, 1995; Heller et al, 1995; Mir et al, 1995), and was also demonstrated to be effective in treatment of accessible tumour nodules of various malignancies in cancer patients (Mir et al, 1998; Serša et al, 1998*a*).

The critical intracellular target for cytotoxicity of both cisplatin and bleomycin is DNA. Bleomycin causes single- and double-stranded breaks in DNA, whereas cytotoxicity of cisplatin is believed to be due to the formation of DNA adducts (Chu, 1994; Mir et al, 1996). Therefore, the cytotoxicity of drugs is dependent on their intracellular concentration and also upon membrane permeability (Chu, 1994; Mir et al, 1996). It was shown that electroporation of cells in vitro potentiates cytotoxicity of bleomycin by several hundred times (Poddevin et al, 1991) and cytotoxicity of cisplatin up to 70 times (Serša et al, 1995). Electroporation of tumour cells in vivo has been demonstrated to potentiate anti-tumour effectiveness of bleomycin and cisplatin several-fold (Mir et al, 1991; Heller et al, 1995; Serša et al, 1995). A high degree of tumour complete responses, as well as tumour cures, were observed at low drug concentrations without systemic toxicity. Studies demonstrating that electroporation increases accumulation of radiolabelled [$^{57}$Co]bleomycin in the cells in vitro (Poddevin et al, 1991) and tumours in vivo (Belehradek et al, 1994) have already been performed.

Whether anti-tumour effectiveness of electrochemotherapy in vivo is due to the increased cisplatin delivery into the tumour cells, caused by electroporation, has been speculated (Serša et al, 1995) but not shown. To answer this question, anti-tumour effectiveness of electrochemotherapy on SA-1 tumours in mice, platinum accumulation in the tumours, and the amount of platinum bound to DNA were measured.

### MATERIALS AND METHODS

### Animals and tumour model

In the experiments, the inbred strain of A/J mice were used, which were purchased from Rudjer Boškovič Institute (Zagreb, Croatia). They were kept at constant room temperature (24°C) with a natural day/night light cycle in a conventional animal colony. Before the experiments, the mice were subjected to an adaptation period of at least 10 days. Female mice in good condition, without fungal or other infections, weighing 20–22 g and 10–12 weeks of age were included in experiments.

*Received 12 March 1998*
*Revised 16 June 1998*
*Accepted 25 June 1998*

*Correspondence to:* G Serša, Institute of Oncology, Department of Tumour Biology, Zaloška 2, SI-1000 Ljubljana, Slovenia

The fibrosarcoma SA-1 tumour model (The Jackson Laboratory, Bar Harbor, ME, USA), syngeneic to A/J mice, was used in the study. Tumour cells were obtained from the ascitic form of the tumours in mice, serially transplanted every 7 days. Solid subcutaneous tumours, located dorsolaterally in mice, were initiated by an injection of $5 \times 10^5$ SA-1 cells in 0.1 ml 0.9% sodium chloride solution. The viability of the cells was over 95% as determined by the Trypan blue dye exclusion test. Six days after transplantation, when the tumours reached approximately 40 $mm^3$ in volume, mice were randomly divided into experimental groups, and subjected to a specific experimental protocol.

### Electrochemotherapy protocol

*cis*-Diamminedichloroplatinum (II) (cisplatin) was obtained from Bristol Myers Squibb (Austria) as a crystalline powder and dissolved in sterile water at a concentration of 1 mg $ml^{-1}$. The final cisplatin solution was freshly prepared in 0.9% sodium chloride each day. Cisplatin at a dose of 4 mg $kg^{-1}$ was injected intravenously into the retro-orbital sinus. Injection volume was 0.0075 ml $g^{-1}$ body weight.

Eight square-wave electric pulses of 1040 V amplitude, with a pulse width of 100 μs and repetition frequency 1 Hz were delivered by two flat, parallel stainless-steel electrodes 8 mm apart (two stainless-steel strips: length 35 mm, width 7 mm with rounded corners), which were placed percutaneously at the opposite margins of the tumour. Good contact between the electrodes and the skin was assured by means of conductive gel. Electric pulses were generated by an electropulsator Jouan GHT 1287 (Saint Herblaine, France). In the electrochemotherapy protocol, mice were treated with electric pulses 3 min after cisplatin injection. All treatments were performed without anaesthesia and were well tolerated by the animals.

Tumour growth was followed by measuring three mutually orthogonal tumour diameters ($e_1$, $e_2$ and $e_3$) with a vernier caliper on each consecutive day. Tumour volumes were calculated by the formula $V = \Pi \times e_1 \times e_2 \times e_3/6$. From these measurements, the arithmetic mean and standard error of the mean were calculated for each experimental group. Tumour growth delay was calculated for each individual tumour by subtracting the doubling time of each tumour from the mean doubling time of the control group and then averaged for each experimental group. Cell kill was calculated from the tumour growth curves using the formula: $\log_{10}$ cell kill = tumour growth delay/3.32 × $T_d$, in which 3.32 is the number of cell doublings per log of growth and $T_d$ is tumour doubling time (Corbett and Valeriote, 1987).

### Platinum determination in the tumours and DNA

To determine platinum content in the tumours and the amount of platinum bound to DNA, mice were sacrificed at various times after treatment with intravenously injected cisplatin and after electrochemotherapy. Tumours (six per group) were excised and removed from the overlying skin.

For platinum determination in whole tumour, each tumour was weighed (tumour weights were approximately 100 mg), placed into a 15-ml graduated polyethylene tube and digested in 1 ml of 65% nitric acid by incubation at 37°C for at least 2 days to obtain a clear solution. Samples were diluted with water to 10 ml before analysis.

For determination of the amount of platinum bound to DNA, each tumour was weighed, minced and treated with collagenase to obtain a single cell suspension. DNA was isolated using a modified salting out method (Miller et al, 1988). Platinum content in the samples was determined by electrothermal atomic absorption spectrometry on a Hitachi Z-8270 Polarized Zeeman Atomic Absorption Spectrophotometer, adjusted to a wavelength of 265.9 nm (Milačič et al, 1997).

### Histology of tumours

The tumours from control, electric pulse, cisplatin- and electrochemotherapy-treated animals were excised 3 days after the treatment. Specimens were fixed immediately in 10% buffered neutral formalin. One tissue block cut through the largest diameter of the tumour was embedded in paraffin, and stained with haematoxylin–eosin by standard method. Slides of six tumours per group were examined in a blind fashion. Tumour necrosis was determined in whole mount tumour section through the largest tumour diameter.

### Statistical analysis

The significance of differences between the mean values of tumour growth delays, degree of necrosis and platinum concentration of the experimental groups was evaluated by a modified *t*-test (Newman Keuls test), after a one-way analysis of variance was performed and fulfilled.

### RESULTS

### Anti-tumour effectiveness

Treatment of animals with cisplatin as a single treatment (4 mg $kg^{-1}$) was ineffective and did not result in significant anti-tumour

**Table 1** Anti-tumour effectiveness of electrochemotherapy on SA-1 tumours in mice

| Therapy | *n* | Tumour doubling time (days) AM±s.e.[a] | Tumour growth delay (days) AM±s.e.[a] |
|---|---|---|---|
| Control | 10 | 1.8 ± 0.1 | – |
| Cisplatin | 10 | 2.0 ± 0.1 | 0.2 ± 0.1 |
| Electric pulses | 10 | 3.7 ± 0.4 | 1.9 ± 0.4 |
| Electrochemotherapy | 10 | 12.1 ± 1.6 | 10.3 ± 1.6 |

[a]Arithmetic mean ± standard error of the mean.

© Cancer Research Campaign 1999

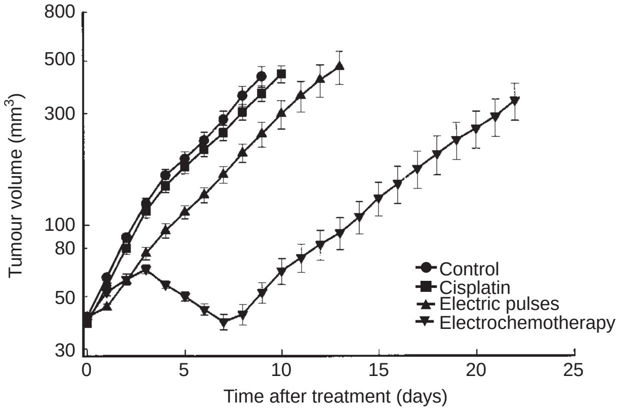

**Figure 1** The anti-tumour effectiveness of electrochemotherapy with cisplatin on fibrosarcoma SA-1 subcutaneous tumours. Mice (ten per group) were treated with cisplatin (4 mg $kg^{-1}$) intravenously and/or with eight electric pulses (1040 V, 100 μs, 1 Hz) 3 min after cisplatin injection. Tumour growth curves represent the arithmetic mean ± standard error of the mean of the tumour volumes measured every 2nd day

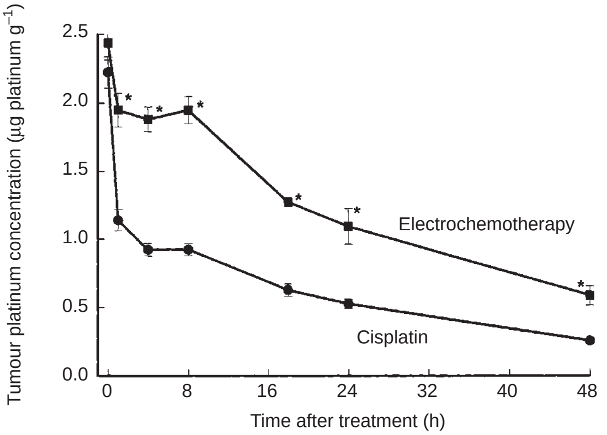

**Figure 2** Platinum concentration at different post-treatment intervals in fibrosarcoma SA-1 tumours treated with cisplatin (4 mg $kg^{-1}$) and electrochemotherapy. Values represent arithmetic mean ± standard error of the mean of six mice per measurement. *$P < 0.05$

effectiveness compared with the control group ($P > 0.05$) (Table 1, Figure 1). The treatment was well tolerated by the animals, body weight was not changed and no mortality was observed (data not shown).

Electric pulses as a single treatment were moderately effective (Table 1, Figure 1). Tumour growth delay was significantly prolonged compared with the control, untreated group ($P < 0.001$). The application of electric pulses had no side-effects and no mortality of the animals was observed (data not shown).

Electrochemotherapy, combining intravenously injecting cisplatin (4 mg $kg^{-1}$) with an application of eight electric pulses to the tumour (1040 V, 100 μs, 1 Hz) 3 min after the injection, was very effective (Table 1, Figure 1). The reduction of tumour volume was already detectable 3 days after the treatment. Thereafter, the tumours gradually decreased in size for up to 7 days, when they reached the pretreatment volume. Tumours were not exulcerated nor had superficial scab. Regrowth of the tumours was observed from the 8th day after the treatment. Their growth rate was approximately the same as in the control groups, but their growth was significantly delayed ($P < 0.001$) compared with control tumours or those treated with cisplatin and electric pulses as single treatment. Treatment with electrochemotherapy resulted in a log cell kill of 0.62 and was more than 20-fold higher than cell kill of cisplatin treatment alone (log cell kill = 0.03), as calculated from tumour growth curves. Electrochemotherapy treatment was well tolerated by the mice, body weight was not changed and no mortality was observed (data not shown).

### Tumour platinum accumulation and amount of platinum bound to DNA

To determine whether the observed anti-tumour effectiveness of electrochemotherapy can be associated with increased cisplatin accumulation in the tumour cells, measurements of platinum content in the tumours and the amount of platinum bound to DNA were performed at different times after electrochemotherapy and cisplatin treatment. Platinum content in the tumour and DNA samples was determined by electrothermal atomic absorption spectrometry (Figure 2, Figure 3). The initial level of platinum in whole tumours was the same in both groups, and wash out of platinum from the tumours was quick. However, significant ($P < 0.008$) differences in the platinum content between the two treatments was observed; after cisplatin treatment and electrochemotherapy, 50% of the initial level was obtained in 1 h and in 18 h respectively. At all time points except $t = 0$, the platinum content in the tumours treated by electrochemotherapy was approximately two times higher than in cisplatin-treated tumours (Figure 2).

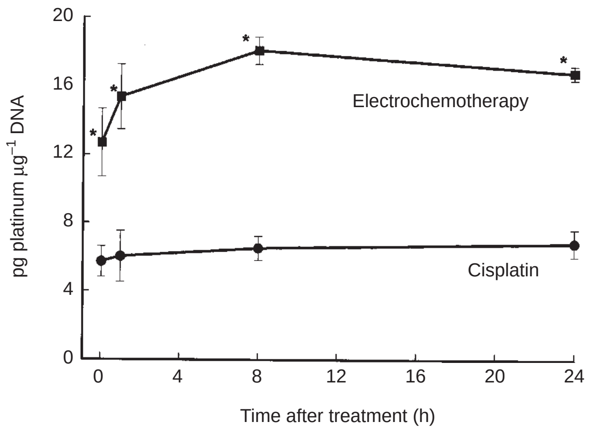

**Figure 3** Amount of platinum bound to DNA of the cells obtained at different post-treatment intervals from tumours treated with cisplatin (4 mg $kg^{-1}$) and electrochemotherapy. Values represent arithmetic mean ± standard error of the mean of six mice per measurement. *$P < 0.05$

Already 3 min after the treatment, two times higher amounts of platinum bound to DNA were detected in the tumours treated by electrochemotherapy than in the tumours treated by cisplatin alone. Thereafter, the amount of platinum bound to DNA in electrochemotherapy-treated tumours increased, reaching the maximum value 8 h after the treatment interval. This is in contrast to cisplatin-treated tumours, in which the amount of platinum bound to DNA was in the same range at all test times (Figure 3).

*© Cancer Research Campaign 1999*

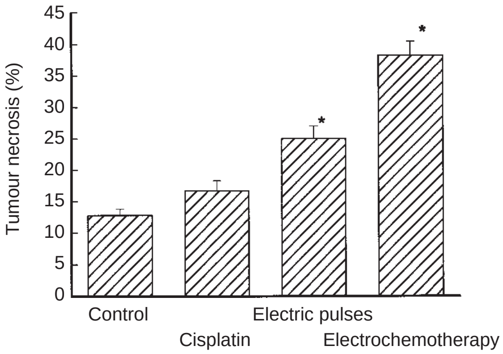

**Figure 4** Percentage of necrosis in the tumours 3 days after treatment with cisplatin (4 mg $kg^{-1}$), electric pulses and electrochemotherapy. Values represent arithmetic mean ± standard error of the mean of six mice per measurement. *$P$<0.05

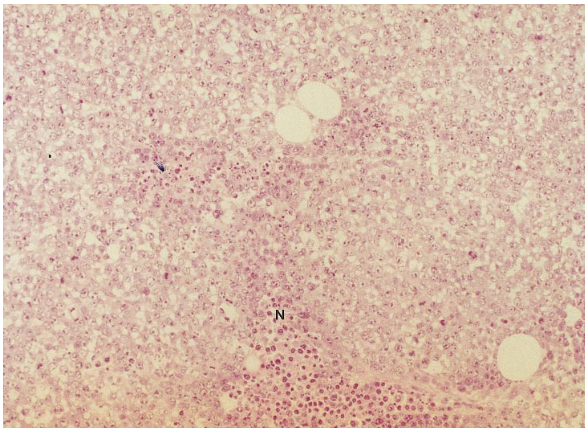

**Figure 5** Fibrosarcoma SA-1 in mouse. Control group. Focal geographic necrosis with pyknotic nuclei (N) (objective 20×)

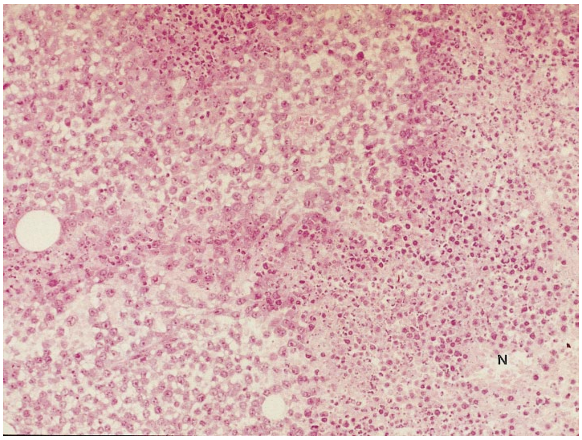

**Figure 6** Fibrosarcoma SA-1 in mouse treated with cisplatin alone. Larger areas of geographic necrosis (N) (objective 20×)

### Degree of necrosis

Necrosis of tumour tissue was evaluated in controls and in all treatment groups 3 days after the beginning of treatment. The degree of necrosis estimated histologically is shown in Figure 4. Cisplatin treatment did not result in significantly increased necrosis compared with controls. However, in tumours exposed to electric pulses and in tumours treated with electrochemotherapy, necrosis was increased significantly ($P$ < 0.001). This degree of necrosis was the highest in tumours treated with electrochemotherapy (38.2% ± 2.2%).

Histologically, necrosis in both control (Figure 5) and cisplatin-treated (Figure 6) groups did not vary except for minor differences in the degree. Focal geographic necroses of tumour cells were characterized mostly by pyknosis, followed by coagulative type of necrosis with preservation of the basic outlines of the tumour cells. In the group treated with electric pulses, necrosis was advanced with large areas of coagulative necrosis with loss of cellular structures (Figure 7). In tumours treated with electrochemotherapy, however, irreversible cell injury was manifested also by confluent areas of cells with distinct changes. Tumour cells were swollen with enlarged vesicular nuclei and solitary or multiple clumped nucleoplasmic condensations in granular background. Perinuclear cytoplasm was condensed, eosinophilic and filled with multiple blebs at the periphery of the cell (Figure 8).

### DISCUSSION

Results of our study demonstrate that anti-tumour effectiveness of electrochemotherapy with cisplatin is associated with increased cisplatin accumulation in the tumours and increased amounts of platinum bound to DNA due to electroporation in vivo. In electrochemotherapy-treated tumours, more than twice the amount of platinum was determined in whole tumours, as well as on DNA compared with cisplatin-treated ones. Because electrochemotherapy produced a more than 20-fold increase in cell kill compared with cisplatin treatment alone, besides electroporation other mechanisms could also be involved in anti-tumour effectiveness of electrochemotherapy.

Electrochemotherapy with cisplatin has already been proven to be effective in treatment of different tumours, as demonstrated in preclinical (Čemažar et al, 1995; Serša et al, 1995, 1997) as well as in clinical studies (Serša et al, 1998*a*). However, it has not been demonstrated yet that the marked increase in anti-tumour effectiveness of cisplatin is due to the increased uptake of cisplatin by the tumour cells after application of electric pulses. Pharmacological studies of electrochemotherapy with belomycin, which is another drug used in electrochemotherapy, have already demonstrated that application of electric pulses to the tumours markedly increases drug accumulation in the tumours (Belehradek et al, 1994). Our study was, thus, undertaken to confirm that cisplatin accumulation in the tumour cells after in vivo electrochemotherapy is also increased.

Cisplatin is a chemotherapeutic drug whose transport through the plasma membrane is restricted (Gately and Howell, 1993). Bypassing the plasma membrane restriction by chemicals or electric pulses (Jekunen et al, 1993; Marverti et al, 1997; Melvik et al, 1986, Serša et al, 1995) could potentiate its anti-tumour effectiveness. Jekunen et al (1993) demonstrated that uptake of [$^3$H]dichloro(ethylendiamine)platinum [II], an analogue of cisplatin, could be increased into human ovarian carcinoma 2008 cells by treating the cells with the plasma membrane-selective detergent digitonin. In addition, stimulation of cisplatin accumulation was also achieved by treating 2008 cells and their resistant subclone C13* with natural polycationic amine spermine

© Cancer Research Campaign 1999 

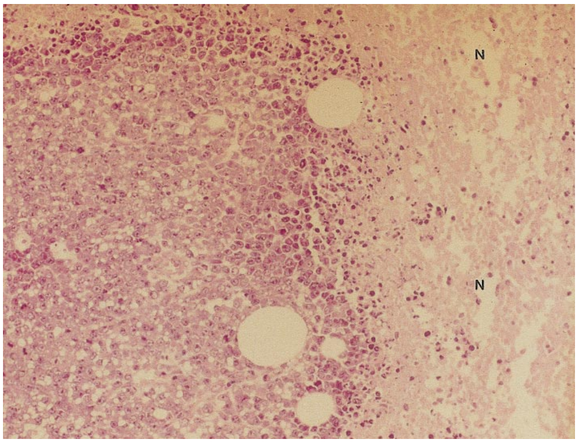

**Figure 7** Fibrosarcoma SA-1 in mouse treated with electric pulses. Areas of advanced coagulative necrosis (N) (objective 20×)

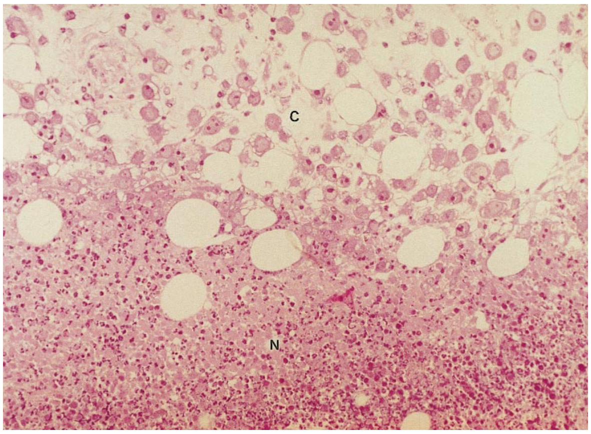

**Figure 8** Fibrosarcoma SA-1 in mouse treated with electrochemotherapy. Areas of necrosis (N) merging with compartment of swollen cells with cytoplasmic condensation and blebbing, vesicular nuclei and chromatin clumps (C) (objective 20×)

(Marverti et al, 1997). In our previous study, we demonstrated that electroporation of B16 melanoma cells in vitro potentiated cisplatin cytotoxicity up to 70 times (Serša et al, 1995). Results of the present study demonstrate that tumour cell kill, platinum content in the tumours and the amount of platinum bound to DNA is increased in electrochemotherapy-treated tumours compared with cisplatin treatment alone. It is well known that DNA platination is the major cytotoxic lesion induced by cisplatin and responsible for cisplatin-induced cell death (Chu, 1994). Therefore, from our results we can conclude that application of electric pulses to tumours in vivo increases the permeability of the plasma membrane of the cells and consequently enables cisplatin to enter the cells and bind to DNA.

Other mechanisms that could contribute to the observed anti-tumour effectiveness of electrochemotherapy could be involvement of the immune system and modification of tumour blood flow due to the application of electric pulses. The role of the immune system in the anti-tumour effectiveness of electrochemotherapy with cisplatin has already been demonstrated (Serša et al, 1997). In that study, we have compared anti-tumour effectiveness of electrochemotherapy with cisplatin in immunocompetent and immunodeficient mice. We found that tumour growth delay in immunocompetent mice was approximately two times longer than in immunodeficient mice, and furthermore a high percentage of tumour cures was obtained in immunocompetent mice in contrast to immunodeficient in which none of the animals were cured. Thus, in the present study, the observed effectiveness of electrochemotherapy could be ascribed also to contribution of the immune system. After electrochemotherapy, less clonogenic tumour cells remain than after cisplatin treatment, therefore the immune system could be more effective in eradicating those cells.

Increased cell kill after electrochemotherapy as well as the observed plateau in the platinum accumulation curve of up to 8 h and the increase in the amount of platinum bound to DNA, with the maximum value at 8 h after the treatment interval, may partly be due to the vascular occlusion in the tumours exposed to electric pulses. As observed in our previous study, besides electroporation, electric pulses can modify tumour blood flow (Serša et al, 1998*b*). Application of electric pulses such as those used in this study reduce tumour blood flow by 60–70% and result in a tumour growth delay of 0.2 days. The changes in blood flow are transient, lasting for 1 h, thereafter tumour blood flow steadily recovers to almost normal levels within 24 h. Having this in mind, the observed retention of platinum in the tumours and increase in DNA platination up to 8 h after the application of electric pulses may be, in addition to electroporation, due to the blood-modifying effect of electric pulses.

Morphological changes in the tumours correlate well with data on anti-tumour effectiveness. Degree of tumour necrosis follows the anti-tumour effectiveness of each therapeutic approach, electrochemotherapy being the most successful. Specifically, morphological changes of tumour cells in tumours treated with electrochemotherapy were different and greater in extent than in tumours treated with cisplatin only.

In conclusion, our study provides evidence that electroporation of tumours in vivo increases uptake of cisplatin in cells. Electrochemotherapy with cisplatin has already been tested in a clinical trial on malignant melanoma, squamous cell carcinoma and basal cell carcinoma (Serša et al, 1998*a*). Therefore, this study further supports this therapy by providing evidence of its underlying mechanisms.

### ACKNOWLEDGEMENT

This work was supported by the Ministry of Science and Technology of the Republic of Slovenia.

### REFERENCES

Belehradek Jr J, Orlowski S, Ramirez LH, Pron G, Poddevin B and Mir LM (1994) Electropermeabilization of cells in tissues assessed by the qualitative and quantitative electroloading of bleomycin. *Biochim Biophys Acta* **1190**: 155–163

Čemažar M, Miklavčič D, Vodovnik L, Jarm T, Rudolf Z, Štabuc B, Čufer T and Serša G (1995) Improved therapeutic effect of electrochemotherapy with cisplatin by intratumoral drug administration and changing of electrode orientation for electropermeabilization on EAT tumors in mice. *Radiol Oncol* **29**: 121–127

Chu G (1994) Cellular responses to cisplatin. *J Biol Chem* **14**: 787–790

Corbett TH and Valeriote FA (1987) Rodent models in experimental chemotherapy. In *Rodent Tumour Models in Experimental Cancer Therapy*. Kallman RF (ed.), pp. 233–247. Pergamon Press: New York

Gately DP and Howell SB (1993) Cellular accumulation of the anticancer agent cisplatin: A review. *Br J Cancer* **67**: 1171–1176

 © Cancer Research Campaign 1999

Heller R, Jaroszeski M, Leo-Messina J, Perrot R, Van Voorhis N, Reintgen D and Gilbert R (1995) Treatment of B16 mouse melanoma with the combination of electropermeabilization and chemotherapy. *Bioelectroch Bioener* **36**: 83–87

Jekunen AP, Shalinsky DR, Hom DK, Albright KD, Health D and Howell SB (1993) Modulation of cisplatin cytotoxicity by permeabilization of the plasma membrane by digitonin in vitro. *Biochem Pharmacol* **45**: 2079–2085

Marveti G, Andrews PA, Piccinini G, Ghiaroni S, Barbieri D and Moruzzi MS (1997) Modulation of cis-diamminedichloroplatinum (II) accumulation and cytotoxicity by spermine in sensitive and resistant human ovarian carcinoma cells. *Eur J Cancer* **33**: 669–675

Melvik JE, Pettersen EO, Gordon PB and Selgen PO (1986) Increase in cis-dichlorodiammineplatinum (II) cytotoxicity upon reversible electropermeabilization of the plasma membrane in cultured human NHIK 3025 cells. *Eur J Cancer Clin Oncol* **22**: 1523–1530

Milačič R, Čemažar M and Serša G (1997) Determination of platinum in tumor tissues after cisplatin therapy by electrothermal atomic absorption spectrometry. *J Pharm Biomed Anal* **16**: 343–348

Miller SA, Dykes DD, Polesky HF (1988) A simple salting out procedure for extracting DNA from human nucleated cells. *Nucleic Acids Res* **16**: 1215

Mir LM, Orlowski S, Belehradek Jr J and Paoletti C (1991) Electrochemotherapy potentiation of antitumor effect of bleomycin by local electric pulses. *Eur J Cancer* **27**: 68–72

Mir LM, Orlowski S, Belehradek Jr J, Teissie J, Rols MP, Serša G, Miklavčič D, Gilbert R and Heller R (1995) Biomedical applications of electric pulses with special emphasis on antitumor electrochemotherapy. *Bioelectroch Bioener* **38**: 203–207

Mir LM, Tounekti O and Orlowski S (1996) Bleomycin: revival of an old drug. *Gen Pharmacol* **27**: 745–748

Mir LM, Glass LF, Seršă G, Teissie J, Domenge C, Miklavčič D, Jaroszeski MJ, Orlowski S, Reintgen DS, Rudolf Z, Belehradek M, Gilbert R, Rols MP, Belehradek Jr J, Bachaud JM, DeConti R, Štabuc B, Čemažar M, Coninx P and Heller R (1998) Effective treatment of cutaneous and subcutaneous malignant tumors by electrochemotherapy. *Br J Cancer* **77**: 2336–2342

Okino M and Mohri H (1987) Effects of a high-voltage electrical impulse and an anticancer drug on in vivo growing tumors. *Jpn J Cancer Res* **78**: 1319–1321

Orlowski S and Mir LM (1993) Cell electropermeabilization: a new tool for biochemical and pharmacological studies. *Biochim Biophys Acta* **1154**: 51–63

Poddevin B, Orlowski S, Belehradek JJr and Mir LM (1991) Very high cytotoxicity of bleomycin introduced into the cytosol of cells in culture. *Biochem Pharmacol* **42**: S67–S75

Serša G, Čemažar M, Miklavčič D and Mir LM (1994) Electrochemotherapy: variable anti-tumor effect on different tumor models. *Bioelectroch Bioener* **35**: 23–27

Serša G, Čemažar M and Miklavčič D (1995) Antitumor effectiveness of electrochemotherapy with cis-diamminedichloroplatinum (II) in mice. *Cancer Res* **55**: 3450–3455

Serša G, Miklavčič D, Čemažar M, Belehradek Jr J, Jarm T and Mir LM (1997) Electrochemotherapy with CDDP on LPB sarcoma: comparison of the anti-tumor effectiveness in immunocompetent and immunodeficient mice. *Bioelectroch Bioener* **43**: 279–283

Serša G, Štabuc B, Čemažar M, Jančar B, Miklavčič D and Rudolf Z (1998*a*) Electrochemotherapy with cisplatin: Potentiation of local cisplatin antitumor effectiveness by application of electric pulses in cancer patients. *Eur J Cancer* **34**: 1213–1218

Serša G, Beravs K, Čemažar M, Miklavčič D and Demšar F (1998*b*) Contrast enhanced MRI assessment of tumor blood volume after application of electric pulses. *Electro Magnetobiol* **17**: 297–304

*© Cancer Research Campaign 1999*
